# Computed Tomography Angiography Markers and Intraluminal Thrombus Morphology as Predictors of Abdominal Aortic Aneurysm Rupture

**DOI:** 10.3390/ijerph192315961

**Published:** 2022-11-30

**Authors:** Emil Marian Arbănași, Adrian Vasile Mureșan, Cătălin Mircea Coșarcă, Eliza Mihaela Arbănași, Raluca Niculescu, Septimiu Toader Voidăzan, Adrian Dumitru Ivănescu, Ioana Hălmaciu, Rareș Cristian Filep, Lucian Mărginean, Shuko Suzuki, Traian V. Chirilă, Réka Kaller, Eliza Russu

**Affiliations:** 1 Doctoral School of Medicine and Pharmacy, George Emil Palade University of Medicine, Pharmacy, Sciences and Technology of Targu Mures, 540142 Targu Mures, Romania; 2 Clinic of Vascular Surgery, Mures County Emergency Hospital, 540136 Targu Mures, Romania; 3 Department of Surgery, George Emil Palade University of Medicine, Pharmacy, Science, and Technology of Targu Mures, 540139 Targu Mures, Romania; 4 Faculty of Pharmacy, George Emil Palade University of Medicine, Pharmacy, Science, and Technology of Targu Mures, 540139 Targu Mures, Romania; 5 Department of Pathophysiology, George Emil Palade University of Medicine, Pharmacy, Science, and Technology of Targu Mures, 540139 Targu Mures, Romania; 6 Department of Epidemiology, George Emil Palade University of Medicine, Pharmacy, Science, and Technology of Targu Mures, 540139 Targu Mures, Romania; 7 Department of Anatomy, George Emil Palade University of Medicine, Pharmacy, Science, and Technology of Targu Mures, 540139 Targu Mures, Romania; 8 Department of Radiology, Mures County Emergency Hospital, 540136 Targu Mures, Romania; 9 Queensland Eye Institute, South Brisbane, QLD 4101, Australia; 10 School of Chemistry & Physics, Queensland University of Technology, Brisbane, QLD 4001, Australia; 11 Australian Institute of Bioengineering & Nanotechnology (AIBN), University of Queensland, St. Lucia, QLD 4072, Australia; 12 Faculty of Medicine, University of Queensland, Herston, QLD 4006, Australia; 13 School of Molecular Sciences, University of Western Australia, Crawley, WA 6009, Australia; 14 Faculty of Medicine, George Emil Palade University of Medicine, Pharmacy, Science, and Technology of Targu Mures, 540139 Targu Mures, Romania

**Keywords:** AAA, imaging markers, ILT, abdominal aortic aneurysm rupture, risk factors, computed tomography angiography

## Abstract

Background: Abdominal aortic aneurysm (AAA) is a complex vascular disease characterized by progressive and irreversible local dilatation of the aortic wall. Currently, the indication for repair is linked to the transverse diameter of the abdominal aorta, using computed tomography angiography imagery, which is one of the most used markers for aneurysmal growth. This study aims to verify the predictive role of imaging markers and underlying risk factors in AAA rupture. Methods: The present study was designed as an observational, analytical, retrospective cohort study and included 220 patients over 18 years of age with a diagnosis of AAA, confirmed by computed tomography angiography (CTA), admitted to Vascular Surgery Clinic of Mures County Emergency Hospital in Targu Mures, Romania, between January 2018 and September 2022. Results: Patients with a ruptured AAA had higher incidences of AH (*p* = 0.006), IHD (*p* = 0.001), AF (*p* < 0.0001), and MI (*p* < 0.0001), and higher incidences of all risk factors (tobacco (*p* = 0.001), obesity (*p* = 0.02), and dyslipidemia (*p* < 0.0001)). Multivariate analysis showed that a high baseline value of all imaging ratios markers was a strong independent predictor of AAA rupture (for all *p* < 0.0001). Moreover, a higher baseline value of DA_max_ (OR:3.91; *p* = 0.001), SA_max_ (OR:7.21; *p* < 0.001), and SLumen_max_ (OR:34.61; *p* < 0.001), as well as lower baseline values of DA_renal_ (OR:7.09; *p* < 0.001), DA_CT_ (OR:12.71; *p* < 0.001), DA_femoral_ (OR:2.56; *p* = 0.005), SA_renal_ (OR:4.56; *p* < 0.001), SA_CT_ (OR:3.81; *p* < 0.001), and SThrombus_max_ (OR:5.27; *p* < 0.001) were independent predictors of AAA rupture. In addition, AH (OR:3.33; *p* = 0.02), MI (OR:3.06; *p* = 0.002), and PAD (OR:2.71; *p* = 0.004) were all independent predictors of AAA rupture. In contrast, higher baseline values of SA_max_/Lumen_max_ (OR:0.13; *p* < 0.001) and ezetimibe (OR:0.45; *p* = 0.03) were protective factors against AAA rupture. Conclusions: According to our findings, a higher baseline value of all imaging markers ratios at CTA strongly predicts AAA rupture and AH, MI, and PAD highly predicted the risk of rupture in AAA patients. Furthermore, the diameter of the abdominal aorta at different levels has better accuracy and a higher predictive role of rupture than the maximal diameter of AAA.

## 1. Introduction

Abdominal aortic aneurysm (AAA) is a complex vascular disease characterized by progressive and irreversible local dilatation of the aortic wall. It is one of the most lethal pathologies, occupying 13th place in the USA [1]. The dilatation may occur along the entire thoracic and abdominal aorta but generally affects the infrarenal part [2,3,4,5]. Among the most important risk factors for AAA are age, smoking habits, hypertension, and family history [6,7].

Although AAA is heavily studied, there are still deficiencies in the early diagnosis of its most feared complication. This downfall appears because most aneurysms are asymptomatic, and are thus discovered by chance [4,5]. Currently, the indication for repair is linked to the transverse diameter of the abdominal aorta, using computed tomography angiography imagery, which is one of the most used markers for aneurysmal growth [8,9].

According to multiple studies, aneurysmal growth of more than 5 cm is associated with a significant risk of rupture [10,11], and a 6-month ultrasonography surveillance is recommended for AAAs with a diameter exceeding 4 cm [12]. The rate of growth is also a significant predictor; hence, a rate of 0.5–1 cm/year is associated with a greater risk of rupture [13].

Numerous diagnostic and prognostic techniques have been presented and evaluated in relation to aneurysmal diameter growth and implicit AAA rupture, but the results have not been consistent and differ from one scientific study to the next. Aortic compliance, mean wall stress (MWS), peak wall stress (PWS), peak wall rupture index (PWRI), and aorta calcifications are among the biomechanical features of the aortic wall that have a role in increasing aneurysmal diameter and the risk of rupture [14,15,16,17,18,19,20]. In the prediction of asymptomatic AAA rupture, Polzer et al. [21] proved that biomechanical rupture risk assessment (BRRA) outperforms maximal aneurysmal diameter.

In a recent study, Jusko et al. [22] revealed that the ratio of the maximum aneurysmal diameter to the aorta diameter at the aneurysmal neck is a better imaging marker for AAA rupture than the maximum diameter (as indicated by the area under the curve (AUC) values using ROC analysis, AUC: 0.783 versus 0.650).

The aims of this study were as follows: (1) to determine the role of imaging markers in AAA rupture risk and (2) to evaluate the risk factors associated with the risk of rupture in AAA patients.

## 2. Materials and Methods

### 2.1. Study Design

The present study was designed as an observational, analytical, retrospective cohort study and included 220 patients over 18 years of age with a diagnosis of AAA, confirmed by computed tomography angiography (CTA), admitted to Vascular Surgery Clinic of Mures County Emergency Hospital in Targu Mures, Romania, between January 2018 and September 2022. Exclusion criteria were as follows: patients with juxta renal AAA, patients with AAA at the level of the iliac and femoral arteries, and saccular AAA.

Regarding the presence of rupture at admission, all patients enrolled in this study were initially divided into two groups named “uAAA” and “rAAA”. The ideal cut-off value for all imaging markers was used to calculate the risk of rupture.

### 2.2. Data Collection

The patient’s age and gender were extracted from the hospital’s electronic database. Regarding comorbidities, the following cardiac pathologies were recorded: arterial hypertension (AH), atrial fibrillation (AF), ischemic heart disease (IHD), history of myocardial infarction (MI), chronic heart failure (CHF), and chronic obstructive pulmonary disease (COPD). Other recorded pathologies included: chronic kidney disease (CKD), peripheral arterial disease (PAD), cerebrovascular accident (CVA), and diabetes mellitus (DM).

### 2.3. CTA Markers

**Figure 1:**
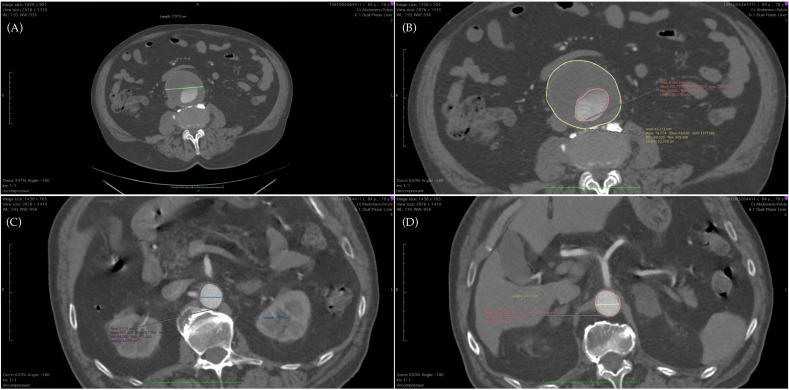
CT angiography: (**A**) maximal diameter of AAA (axial section); (**B**) surface of AAA at maximal diameter (yellow) and surface of lumen (orange); (**C**) diameter and surface of aorta at renal level; and (**D**) diameter and surface of aorta at celiac trunk level.

CTA Markers were determined from the measurements of the abdominal aorta at different levels, and the ratios were calculated using the equations as seen in Table 1 and Figure 1. Moreover, intraluminal thrombus (ILT) morphology was divided into four categorizations: eccentric (anterior, posterior, and lateral) and concentric.

### 2.4. Study Outcomes

The primary endpoint was the risk of AAA rupture. The outcome was stratified based on the optimal cut-off value of imaging markers.

### 2.5. Statistical Analysis

For statistical analysis, SPSS for Mac OS version 28.0.1.0 was utilized (SPSS, Inc., Chicago, IL, USA). To analyze the correlations of the ratios with categorical factors, chi-square tests were performed. T-Student or Mann–Whitney tests were used to assess differences in continuous variables. The receiver operating characteristic (ROC) curve analysis was used to assess the prediction capability and to set the cut-off values for all imaging indicators. The Youden index was utilized to calculate the optimal imaging marker cut-off values (Youden Index = Sensitivity + Specificity 1, ranging from 0 to 1). A multivariate logistic regression analysis with factors of *p* < 0.1 was performed to establish independent predictors of AAA rupture.

## 3. Results

During the studied period, 220 patients were enrolled, from whom 173 patients (78.63%) were diagnosed with AAA without rupture, and 47 patients (21.37%) were diagnosed with ruptured AAA. Of the patients, 123 were male (55.91%), and the mean age was 71.68 ± 9.78 (47–95). The rest of the recorded variables are presented in Table 2.

Patients with rAAA had higher incidences of AH (*p* = 0.006), IHD (*p* = 0.001), AF (*p* < 0.0001), and MI (*p* < 0.0001) and higher incidences of all risk factors (tobacco (*p* = 0.001), obesity (*p* = 0.02), and dyslipidemia (*p* < 0.0001)) as seen in Table 2.

Regarding the CTA markers, patients in the rAAA group had higher values of DA_max_ (*p* = 0.003), SA_max_ (*p* < 0.0001), SLumen_max_ (*p* < 0.0001), DA_max_/A_renal_ (*p* < 0.0001), DA_max_/A_CT_ (*p* < 0.0001), DA_max_/A_femoral_ (*p* = 0.01), SA_max_/A_renal_ (*p* < 0.0001), SA_max_/A_CT_ (*p* < 0.0001), SA_max_/A_femoral_ (*p* < 0.0001), SA_max_/Thrombus_max_ (*p* < 0.0001), SLumen_max_/Thrombus_max_ (*p* < 0.0001), as well lower values of DA_renal_ (*p* < 0.0001), DA_CT_ (*p* < 0.0001), DA_femoral_ (*p* = 0.005), SA_renal_ (*p* < 0.0001), SA_CT_ (*p* < 0.0001), and SA_max_/Lumen_max_ (*p* < 0.0001). In terms of ILT morphology, there was a higher incidence of anterior-eccentric distribution (*p* = 0.003), as well as a lower incidence of concentric distribution (*p* = 0.03) in the rAAA group.

**Figure 2:**
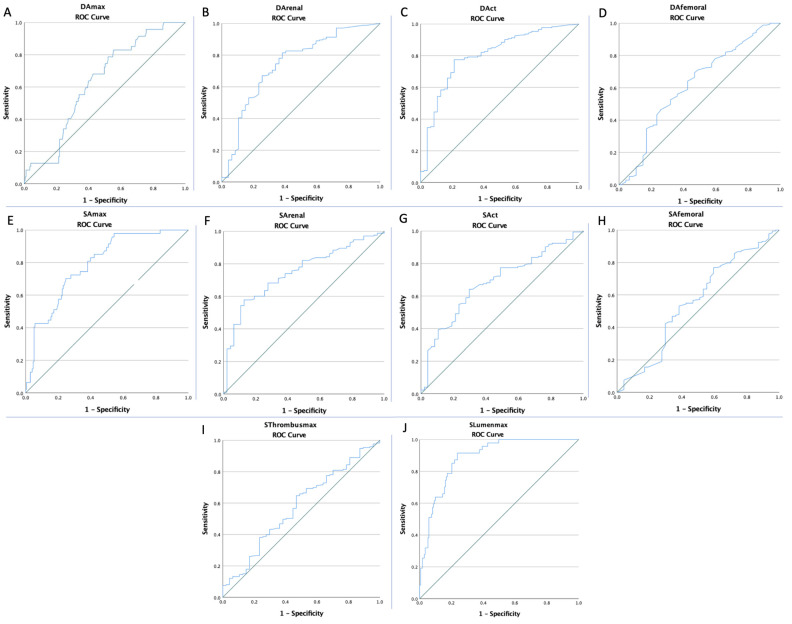
The ROC curve analysis concerning AAA rupture for the (**A**) DA_max_ (AUC: 0.630; *p* = 0.006), (**B**) DA_renal_ (AUC: 0.744; *p* < 0.0001), (**C**) DA_CT_ (AUC: 0.802; *p* < 0.0001), (**D**) DA_femoral_ (AUC: 0.620; *p* = 0.01), (**E**) SA_max_ (AUC: 0.789; *p* < 0.0001), (**F**) SA_renal_ (AUC: 0.746; *p* < 0.0001), (**G**) SA_CT_ (AUC: 0.684; *p* < 0.0001), (**H**) SA_femoral_ (AUC: 0.556; *p* = 0.24), (**I**) SLumen_max_ (AUC: 0.887; *p* < 0.0001), and (**J**) SThrombus_max_ (AUC: 0.577; *p* = 0.10).

**Figure 3:**
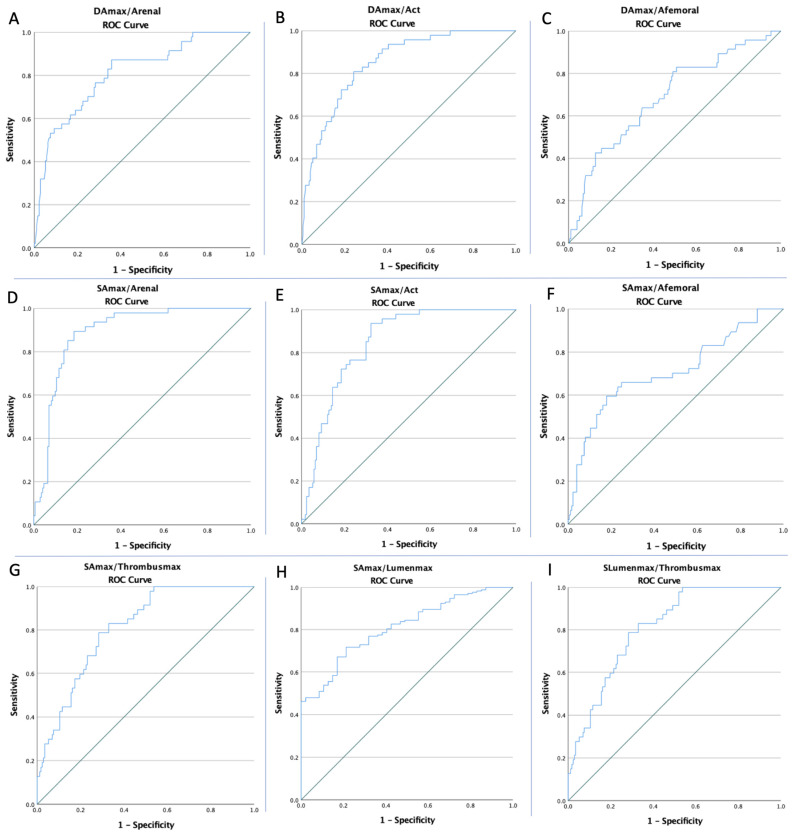
The ROC curve analysis concerning AAA rupture for the (**A**) DA_max_/A_renal_ (AUC: 0.810; *p* < 0.0001), (**B**) DA_max_/A_CT_ (AUC: 0.850; *p* < 0.0001), (**C**) DA_max_/A_femoral_ (AUC: 0.687; *p* < 0.0001), (**D**) SA_max_/A_renal_ (AUC: 0.890; *p* < 0.0001), (**E**) SA_max_/A_CT_ (AUC: 0.846; *p* < 0.0001), (**F**) SA_max_/A_femoral_ (AUC: 0.709; *p* < 0.0001), (**G**) SA_max_/Thrombus_max_ (AUC: 0.809; *p* < 0.0001), (**H**) SA_max_/Lumen_max_ (AUC: 0.809; *p* < 0.0001), and (**I**) SLumen_max_/Thrombus_max_ (AUC: 0.809; *p* < 0.0001).

The ROC curves of all imaging markers were created to determine whether the baseline of these markers was predictive of AAA rupture (Figure 2 and Figure 3). The optimal cut-off value obtained from Youden’s index, areas under the curve (AUC), and the predictive accuracy of the markers are listed in Table 3.

The risk of AAA rupture was further analyzed after dividing the patients into paired groups according to the optimal cut-off value of imaging markers. Moreover, as seen in Table 4, there was a higher incidence of AAA rupture risk for all the imaging markers, with exceptions for DA_renal_, DA_CT_, SA_renal_, SA_CT_, SA_max_/SLumen_max_, where lower incidences of AAA rupture were reported.

A multivariate analysis was used to determine the association between the imaging markers, underlying risk factors, and AAA rupture risk. A high baseline value of all imaging ratio markers was a strong independent predictor of AAA rupture (for all *p* < 0.0001). Moreover, as shown in Table 5, higher baseline values of DA_max_ (OR:3.91; *p* = 0.001), SA_max_ (OR:7.21; *p* < 0.001), and SLumen_max_ (OR:34.61; *p* < 0.001), as well as lower baseline values of DA_renal_ (OR:7.09; *p* < 0.001), DA_CT_ (OR:12.71; *p* < 0.001), DA_femoral_ (OR:2.56; *p* = 0.005), SA_renal_ (OR:4.56; *p* < 0.001), SA_CT_ (OR:3.81; *p* < 0.001), and SThrombus_max_ (OR:5.27; *p* < 0.001) were independent predictors of AAA rupture. Furthermore, AH (OR:3.33; *p* = 0.02), MI (OR:3.06; *p* = 0.002), PAD (OR:2.71; *p* = 0.004), and anterior-eccentric morphology of ILT (OR:2.84; *p* = 0.004) were all independent predictors of AAA rupture. In contrast, the higher baseline values of SA_max_/Lumen_max_ (OR:0.13; *p* < 0.001) and concentric morphology of ILT (OR:0.21; *p* = 0.03) were protective factors against AAA rupture (Table 5).

## 4. Discussion

The primary outcome of this research is that CT angiography imaging markers are highly predictive of AAA rupture risk. As seen in Table 5, cardiovascular diseases (AH, MI, and PAD) and the distribution of intraluminal thrombus predict AAA rupture. To the best of our knowledge, this is the first conducted research to evaluate the diameter of the abdominal aorta at different levels, intraluminal thrombus distribution, specific imaging markers, and the risk of AAA rupture.

AAA is a serious public health issue worldwide, with a high incidence ranging from 1.3% to 12.5% depending on sex. High mortality rates exist in the case of a ruptured AAA [23,24]. In 2019, 172,000, deaths were reported in patients who presented with a ruptured AAA, indicating a rise of more than 80% over the previous 20 years [25,26].

Cardiovascular diseases and risk factors such as smoking, and obesity are among the possible causes involved in the growth and risk of AAA rupture. Similar to our finding, numerous research [27,28,29] considers the occurrence of AH to also be a risk factor. Furthermore, several studies have shown that the presence of PAD, IHD, a history of MI, and coronary artery disease is associated with the presence of AAA and an increased risk of AAA rupture [30,31,32,33,34].

The role of the intraluminal thrombus in the case of AAA evolution has been extensively debated in the specialized literature, with mixed results. Some studies emphasize the protective role of the thrombus [35,36,37,38], while others show the involvement of the thrombus in increasing aneurysmal diameter, weakening the aortic wall, and increasing the risk of rupture [39,40,41]. As shown in Table 5, the circumferential arrangement of the intraluminal thrombus has a protective effect in the case of AAA rupture (OR: 6.41, *p* < 0.001).

Zhu et al. [42] demonstrated in the multivariate analysis that the basal diameter of the AAA (*p* = 0.001) and the presence of ILT (*p* = 0.02) are positively associated with the growth rate of the aneurysmal diameter. Additionally, in the systematic review and meta-analysis published by Singh et al. [18], which included eight studies and a total of 672 patients, they discovered an increase in ILT volume in patients with a ruptured AAA (*p* = 0.005). Recently, Kontopodis et al. [43] demonstrated that the relative volume of the ILT (37.5% vs. 73.5%; *p* = 0.004) and maximum thickness (14.5 mm vs. 28 mm; *p* = 0.001) presented lower values in patients with AAA rupture. Moreover, an ILT volume greater than 51.6% correlates with severe adverse events, as shown in a multivariate analysis (HR: 2.90; *p* = 0.04) in a study published by Ding et al. [44], which studied the case of 184 patients with AAA following endovascular aneurysm repair. In contrast, the descriptive review published by Boyd et al. [45] emphasized the protective role of ILT by reducing wall stress.

Maximum aneurysmal diameter is the most often utilized imaging marker in evaluating the risk of AAA rupture [42,46,47,48,49,50,51,52,53,54,55]. The current guidelines of the European Society of Vascular and Endovascular Surgery (ESVES) propose a surgical or endovascular resolution of AAA with dimensions higher than 5.5 cm in male patients and less than 5 cm in female patients [56]. In addition, Choksy et al. [57] and Hall et al. [58] observed an AAA rupture rate of 7.4% in AAAs with a diameter of less than 6 cm, and 7.5% in patients with AAAs with a diameter of less than 5 cm. As a result, it is required to study and suggest novel imaging methods for AAA rupture prognosis.

Similar to our study, Siika et al. [59] discovered that a greater diameter of the aortic lumen area (*p* = 0.02) and a lower ILT area ratio (*p* = 0.03) are related to an increased risk of AAA rupture. Chung et al. [16] indicated that aneurysmal sac analysis provides us with useful information in stratifying AAA with risk, and hence, PWS (*p* = 0.003) and MWS (*p* = 0.02) have higher values in the group with unstable AAA.

This study complements the studies carried out by Jusko et al. [22], Fillinger et al. [60], Di Martino et al. [61], and Kimura et al. [62], who demonstrated that the geometric analysis of the AAA provides valuable information, with a predictive role superior to that of the maximum diameter in the case of the risk of AAA rupture.

According to our study, among the imaging markers analyzed in the multivariate analysis, the high basal values of SA_max_/A_renal_ (OR:37.01; *p* < 0.001), SLumen_max_ (OR:34.61; *p* < 0.001), and SA_max_/A_CT_ (OR:30.64; *p* < 0.001), show the greatest predictive role of AAA rupture, and are superior to DA_max_ (OR:3.92; *p* = 0.001) or SA_max_ (OR:7.12; *p* < 0.001), as seen in Table 5.

The strength of this study is represented by the multitude of imaging markers analyzed, for which the predictive roles were demonstrated. However, despite the significant results and the increased sensitivity and specificity of the analyzed markers, our study has numerous limitations. Firstly, it is a retrospective, monocentric study. Secondly, we did not follow the evolution of the patients and did not record the type of intervention for each patient. Moreover, given the retrospective design, we did not have data on chronic medication before the hospitalization of the patients. In the future, we propose to analyze the proposed markers in a study in which we will follow their predictive role in AAA growth. Additional studies are also needed to validate the results obtained in this study.

## 5. Conclusions

According to our findings, a higher baseline value of all imaging marker ratios at CTA strongly predicts AAA rupture. In addition, AH, MI, and PAD highly predict the risk of rupture in AAA patients. Furthermore, the diameter of the abdominal aorta at different levels has better accuracy and a higher predictive role of rupture than the maximal diameter of AAA. Given the significant risk of mortality in cases with ruptured AAA and the ease with which the measurements for the imaging markers proposed in this study are determined, the measurements can be used to identify patients at high risk of rupture, improve patient care, and develop predictive patterns.

## Figures and Tables

**Table 1:** Imaging markers definitions.

Markers	Definition
DA_max_	maximum diameter of the AAA
DA_renal_	diameter of the aorta at renal level
DA_CT_	diameter of the aorta at celiac trunk level
DA_femoral_	diameter of the femoral artery
SA_max_	surface of the AAA at maximum diameter
SA_renal_	surface of the aorta at renal level
SA_CT_	surface of the aorta at celiac trunk level
SA_femoral_	surface of the femoral artery
SLumen_max_	surface of the lumen at maximum diameter of the AAA
SThrombus_max_	surface of the thrombus at maximum diameter of the AAA
DA_max_/A_renal_	$\frac{\mathrm{maximum}\;\mathrm{diameter}\;\mathrm{of}\;\mathrm{the}\;\mathrm{AAA}}{\mathrm{diameter}\;\mathrm{of}\;\mathrm{the}\;\mathrm{aorta}\;\mathrm{at}\;\mathrm{renal}\;\mathrm{level}}$
DA_max_/A_CT_	$\frac{\mathrm{maximum}\;\mathrm{diameter}\;\mathrm{of}\;\mathrm{the}\;\mathrm{AAA}}{\mathrm{diameter}\;\mathrm{of}\;\mathrm{the}\;\mathrm{aorta}\;\mathrm{at}\;\mathrm{celiac}\;\mathrm{trunk}\;\mathrm{level}}$
SA_max_/A_renal_	$\frac{\mathrm{surface}\;\mathrm{of}\;\mathrm{the}\;\mathrm{AAA}\;\mathrm{at}\;\mathrm{maximum}\;\mathrm{diameter}}{\mathrm{surface}\;\mathrm{of}\;\mathrm{the}\;\mathrm{aorta}\;\mathrm{at}\;\mathrm{renal}\;\mathrm{level}}$
SA_max_/A_CT_	$\frac{\mathrm{surface}\;\mathrm{of}\;\mathrm{the}\;\mathrm{AAA}\;\mathrm{at}\;\mathrm{maximum}\;\mathrm{diameter}}{\mathrm{surface}\;\mathrm{of}\;\mathrm{the}\;\mathrm{aorta}\;\mathrm{at}\;\mathrm{celiac}\;\mathrm{trunk}\;\mathrm{level}}$
SA_max_/Lumen_max_	$\frac{\mathrm{surface}\;\mathrm{of}\;\mathrm{the}\;\mathrm{AAA}\;\mathrm{at}\;\mathrm{maximum}\;\mathrm{diameter}}{\mathrm{surface}\;\mathrm{of}\;\mathrm{the}\;\mathrm{lumen}\;\mathrm{at}\;\mathrm{maximum}\;\mathrm{diameter}\;\mathrm{of}\;\mathrm{the}\;\mathrm{AAA}}$
SLumen_max_/Thrombus_max_	$\frac{\mathrm{surface}\;\mathrm{of}\;\mathrm{the}\;\mathrm{lumen}\;\mathrm{at}\;\mathrm{maximum}\;\mathrm{diameter}\;\mathrm{of}\;\mathrm{the}\;\mathrm{AAA}}{\mathrm{surface}\;\mathrm{of}\;\mathrm{the}\;\mathrm{thrombus}\;\mathrm{at}\;\mathrm{maximum}\;\mathrm{diameter}\;\mathrm{of}\;\mathrm{the}\;\mathrm{AAA}}$

**Table 2:** The baseline characteristics data of all patients, divided according to the AAA rupture risk.

Variables	All Patients n = 220	uAAA n = 173	rAAA n = 47	*p* Value (OR; CI 95%)
Age mean ± SD (min–max)	71.68 ± 9.78 (47–95)	71.58 ± 10.13 (47–95)	72.06 ± 8.44 (55–88)	0.74
Male/Female sex no. (%)	123 (55.91%) 97 (44.09%)	95 (54.91%) 78 (45.09%)	28 (59.57%) 19 (40.43%)	0.14 (1.45; 0.87–2.42)
Comorbidities and Risk factors, no. (%)				
AH, no. (%)	175 (79.54%)	134 (77.45%)	41 (87.23%)	0.006 (2.30; 1.26–4.19)
IHD, no. (%)	159 (72.27%)	120 (69.36%)	39 (82.97%)	0.001 (2.32; 1.38–3.89)
AF, no. (%)	62 (28.18%)	48 (27.74%)	14 (29.78%)	<0.0001 (3.23; 1.90–5.48)
CHF, no. (%)	73 (33.18%)	57 (32.94%)	16 (34.04%)	0.77 (1.09; 0.60–1.98)
MI, no. (%)	44 (20%)	27 (15.6%)	17 (36.17%)	<0.0001 (3.16; 1.83–5.44)
DM, no. (%)	66 (30%)	52 (30.05%)	14 (29.78%)	0.25 (1.37; 0.79–2.35)
CKD, no. (%)	33 (15%)	25 (14.45%)	8 (17.02%)	0.74 (1.11; 0.58–2.10)
COPD, no. (%)	24 (10.9%)	17 (9.82%)	7 (14.89%)	0.74 (1.11; 0.58–2.10)
PAD, no. (%)	103 (46.81%)	71 (41.04%)	32 (68.08%)	0.64 (1.14; 0.63–2.06)
CVA, no. (%)	64 (29.09%)	46 (26.58%)	18 (38.29%)	0.74 (1.11; 0.58–2.10)
Tobacco, no. (%)	58 (26.36%)	41 (23.69%)	17 (36.17%)	0.001 (2.55; 1.46–4.46)
Obesity, no. (%)	50 (22.72%)	31 (17.91%)	19 (40.42%)	0.02 (1.90; 1.10–3.28)
Dyslipidemia, no. (%)	39 (17.72%)	30 (17.34%)	9 (19.14%)	<0.0001 (5.27; 3.07–9.02)
Computed Tomography Angiography Markers, median [Q1–Q3]				
DA_max_	6.75 [5.71–8.15]	6.42 [5.64–8.1]	7.63 [6.31–8.41]	0.003
DA_renal_	2.15 [1.64–2.50]	2.26 [1.88–2.56]	1.59 [1.27–2.04]	<0.0001
DA_CT_	2.38 [1.83–2.86]	2.56 [2.13–2.95]	1.78 [1.53–2.04]	<0.0001
DA_femoral_	0.93 [0.78–1.12]	0.95 [0.80–1.13]	0.84 [0.72–0.98]	0.005
SA_max_	53.7 [39.51–74.26]	48.3 [36.01–69.94]	75.73 [57.83–91.78]	<0.0001
SA_renal_	4.95 [3.89–5.91]	5.29 [4.34–6.31]	3.89 [3.53–4.79]	<0.0001
SA_CT_	5.33 [4.44–6.69]	5.55 [4.69–6.95]	4.59 [4.01–5.42]	<0.0001
SA_femoral_	1.19 [0.87–1.61]	1.24 [0.89–1.59]	1.15 [0.81–1.98]	0.12
SLumen_max_	28.98 [13.01–44.77]	23.47 [10.42 = 35.31]	55.31 [42.79–65.64]	<0.0001
SThrombus_max_	21.81 [13.38–34.23]	22.9 [14.21–34.42]	16.41 [11.72–29.09]	0.052
DA_max_/A_renal_	3.29 [2.49–4.37]	3.04 [2.34–3.92]	4.60 [3.86–5.41]	<0.0001
DA_max_/A_CT_	2.92 [2.26–3.97]	2.71 [2.03–3.39]	4.29 [3.45–5.15]	<0.0001
DA_max_/A_femoral_	7.40 [5.83–9.06]	7.10 [5.46–8.81]	8.81 [6.89–10.36]	0.01
SA_max_/A_renal_	10.65 [7.47–16.12]	8.89 [7.03–12.98]	18.9 [15.9–21.05]	<0.0001
SA_max_/A_CT_	9.41 [6.8–14.66]	7.79 [6.38–12.02]	15.6 [12.89–20.76]	<0.0001
SA_max_/A_femoral_	42.44 [26.79–72.19]	38.61 [25.73–57.67]	82.22 [32.17–106.3]	<0.0001
SA_max_/Lumen_max_	1.72 [1.35–2.89]	2.06 [1.49–3.89]	1.32 [1.20–1.56]	<0.0001
SA_max_/Thrombus_max_	2.37 [1.52–3.80]	1.94 [1.34–3.007]	4.04 [2.75–5.89]	<0.0001
SLumen_max_/Thrombus_max_	1.37 [0.52–2.80]	0.94 [0.34–2.007]	3.04 [1.75–4.89]	<0.0001
Intraluminal Thrombus Morphology, no. (%)				
Posterior-Eccentric	77 (35%)	61 (35.26%)	16 (34.04%)	0.87 (0.94; 0.48–1.86)
Anterior-Eccentric	49 (22.27%)	31 (17.92%)	18 (38.3%)	0.003 (2.84; 1.40–5.75)
Lateral-Eccentric	62 (28.18%)	49 (28.32%)	13 (27.66%)	0.92 (0.96; 0.47–1.98)
Concentric	32 (14.55%)	30 (17.34%)	2 (4.26%)	0.03 (0.21; 0.04–0.92)

AH = arterial hypertension; IHD = ischemic heart disease; AF = atrial fibrillation; CHF = chronic heart failure; MI = myocardial infarction; CKD = chronic kidney disease; COPD = chronic obstructive pulmonary disease; PAD = peripheral arterial disease; CVA = cerebrovascular accident.

**Table 3:** The AUC of the ROC curve, 95% confidence interval, sensitivity, and specificity of the imaging markers.

Variables	Cut-Off	AUC	Std. Error	95% CI	Sensitivity	Specificity	*p* Value
AAA Rupture							
DA_max_	6.11	0.630	0.041	0.549–0.711	83%	44.5%	0.006
DA_renal_	1.73	0.744	0.043	0.660–0.828	81.5%	61.7%	<0.0001
DA_CT_	2.08	0.802	0.037	0.730–0.875	77.5%	78.7%	<0.0001
DA_Femoral_	0.84	0.620	0.049	0.524–0.716	71.1%	51.1%	0.01
SA_max_	65.16	0.789	0.034	0.723–0.856	70.2%	75.1%	<0.0001
SA_renal_	4.72	0.746	0.037	0.673–0.819	63.6%	72.3%	<0.0001
SA_CT_	5.09	0.684	0.042	0.603–0.766	64.2%	68.1%	<0.0001
SA_femoral_	0.91	0.556	0.051	0.457–0.655	73.4%	40.4%	0.24
SLumen_max_	35.94	0.887	0.023	0.842–0.932	91.5%	76.3%	<0.0001
SThrombus_max_	17.48	0.577	0.047	0.485–0.669	64.7%	51.1%	0.10
DA_max_/A_renal_	3.27	0.810	0.036	0.740–0.880	87.2%	64.2%	<0.0001
DA_max_/ACT	3.07	0.850	0.029	0.794–0.907	80.9%	75.7%	<0.0001
DA_max_/A_femoral_	6.78	0.687	0.044	0.600–0.773	83%	49.1%	<0.0001
SA_max_/A_renal_	14.27	0.890	0.023	0.844–0.935	89.4%	81.5%	<0.0001
SA_max_/A_CT_	9.85	0.846	0.027	0.793–0.898	93.6%	67.6%	<0.0001
SA_max_/A_femoral_	58.19	0.709	0.047	0.618–0.800	66%	75.1%	<0.0001
SA_max_/Lumen_max_	1.57	0.809	0.031	0.749–0.870	71.7%	78.7%	<0.0001
SA_max_/Thrombus_max_	2.75	0.809	0.031	0.749–0.870	78.7%	71.7%	<0.0001
SLumen_max_/Thrombus_max_	1.75	0.809	0.031	0.749–0.870	78.7%	71.7%	<0.0001

**Table 4:** Univariate analysis of imaging markers and risk of AAA rupture.

	rAAA		rAAA
Low-DA_max_ vs. High-DA_max_	14/144 (9.72%) vs. 33/76 (43.42%) *p* < 0.0001	Low-DA_max_/A_renal_ vs. High-DA_max_/A_renal_	6/117 (5.13%) vs. 41/103 (39.81%) *p* < 0.0001
High-DA_renal_ vs. Low-DA_renal_	18/159 (11.32%) vs. 29/61 (47.5%) *p* < 0.0001	Low-DA_max_/A_CT_ vs. High-DA_max_/A_CT_	3/120 (2.5%) vs. 44/100 (44%) *p* < 0.0001
High-DA_CT_ vs. Low-DA_CT_	10/144 (6.94%) vs. 37/76 (48.68%) *p* < 0.0001	Low-DA_max_/A_Femoral_ vs. High-DA_max_/A_Femoral_	3/120 (2.5%) vs. 44/100 (44%) *p* < 0.0001
Low-DA_Femoral_ vs. High-DA_Femoral_	23/146 (15.7%) vs. 24/74 (32.43%) *p* = 0.005	Low-SA_max_/A_renal_ vs. High-SA_max_/A_renal_	10/134 (7.46%) vs. 37/86 (43.02%) *p* < 0.0001
Low-SA_max_ vs. High-SA_max_	14/144 (9.72%) vs. 33/76 (43.42%) *p* < 0.0001	Low-SA_max_/A_CT_ vs. High-SA_max_/A_CT_	3/120 (2.5%) vs. 44/100 (44%) *p* < 0.0001
High-SA_renal_ vs. Low-SA_renal_	13/123 (10.5%) vs. 34/97 (35.05%) *p* < 0.0001	Low-SA_max_/A_Femoral_ vs. High-SA_max_/A_Femoral_	16/146 (10.96%) vs. 31/74 (41.9%) *p* < 0.0001
Low-SA_CT_ vs. High-SA_CT_	3/120 (2.5%) vs. 44/100 (44%) *p* < 0.0001	Low-SA_max_/SLumen_max_ vs. High-SA_max_/SLumen_max_	35/84 (41.67%) vs. 12/136 (8.82%) *p* < 0.0001
Low-SLumen_max_ vs. High-SLumen_max_	4/136 (2.94%) vs. 43/84 (51.19%) *p* < 0.0001	Low-SLumen_max_/Thrombus_max_ vs. High-SLumen_max_/Thrombus_max_	10/134 (7.46%) vs. 37/86 (43.02%) *p* < 0.0001
Low-SA_max_/Thrombus_max_ vs. High-SA_max_/Thrombus_max_		10/134 (7.46%) vs. 37/86 (43.02%) *p* < 0.0001	

**Table 5:** Multivariate analysis for predictors AAA rupture.

	rAAA		
Variables	OR	95% CI	*p* Value
Comorbidities and Risk Factors			
AH	3.33	1.13–9.86	0.02
MI	3.06	1.48–6.31	0.002
PAD	2.71	1.38–5.33	0.004
Tobacco	1.41	0.70–2.86	0.33
Obesity	0.52	0.22–1.26	0.15
Intraluminal Thrombus Morphology			
Anterior-Eccentric	2.84	1.40–5.75	0.004
Concentric	0.21	0.04–0.92	0.03
Computed Tomography Angiography Markers			
High-DA_max_	3.91	1.72–8.85	0.001
Low-DA_renal_	7.09	3.51–14.32	<0.001
Low-DA_CT_	12.71	5.80–27.85	<0.001
Low-DA_femoral_	2.56	1.32–4.95	0.005
High-SA_max_	7.12	3.49–14.55	<0.001
Low-SA_renal_	4.56	2.24–9.29	<0.001
Low-SA_CT_	3.81	1.92–7.59	<0.001
High-SLumen_max_	34.61	11.72–102.20	<0.001
Low-SThrombus_max_	5.27	3.07–9.02	<0.001
High-DA_max_/A_renal_	12.23	4.91–30.43	<0.001
High-DA_max_/A_CT_	11.93	5.03–28.32	<0.001
High-DA_max_/A_femoral_	4.60	2.03–10.41	<0.001
High-SA_max_/A_renal_	37.01	13.56–100.96	<0.001
High-SA_max_/ACT	30.64	9.11–102.97	<0.001
High-SA_max_/A_femoral_	5.85	2.92–11.73	<0.001
High-SA_max_/Lumen_max_	0.13	0.06–0.28	<0.001
High-SA_max_/Thrombus_max_	9.36	4.32–20.28	<0.001
High-SLumen_max_/Thrombus_max_	9.36	4.32–20.28	<0.001

AH = arterial hypertension; MI = myocardial infarction; PAD = peripheral arterial disease.

## Data Availability

Not applicable.
